# Genome-wide identification and comprehensive analysis of the NAC transcription factor family in *Sesamum indicum*

**DOI:** 10.1371/journal.pone.0199262

**Published:** 2018-06-21

**Authors:** Yujuan Zhang, Donghua Li, Yanyan Wang, Rong Zhou, Linhai Wang, Yanxin Zhang, Jingyin Yu, Huihui Gong, Jun You, Xiurong Zhang

**Affiliations:** 1 Key Laboratory of Biology and Genetic Improvement of Oil Crops, Ministry of Agriculture Oil Crops Research Institute of the Chinese Academy of Agricultural Sciences, Wuhan, China; 2 Cotton Research Center, Shandong Academy of Agricultural Sciences, Jinan, China; Huazhong University of Science and Technology, CHINA

## Abstract

The NAM, ATAF1/2, and CUC2 (NAC) family constitutes a large family of plant-specific transcription factors, involved in many aspects of physiological processes and a variety of abiotic stresses. There is little information concerning the NAC family in *Sesamum indicum*. In this study, 87 sesame NAC genes were identified and phylogenetically clustered into 12 groups with *Arabidopsis* NAC genes. A total of 83 *SiNAC* genes were distributed non-randomly on the 16 linkage groups in sesame. Four and 49 *SiNAC*s were found to be tandemly and segmentally duplicated, respectively. Expression profiles of *SiNAC* genes in different tissues (root, stem, leaf, flower, seed, and capsule) and in response to drought and waterlogging stresses by using RNA-seq data demonstrated that 23 genes were highly expressed in all tissues, 18 and 31 *SiNAC*s respond strongly to drought and waterlogging stresses, respectively. In addition, the expression of 30 *SiNAC* genes distributed in different subgroups was analyzed with quantitative real-time RT-PCR under cold, osmotic, and salt stresses, revealed that their expression patterns vary in response to abiotic stresses. *SiNAC* genes displayed diverse expression patterns among the different tissues and stress treatments, suggested that their contribution to plant growth and development in sesame and multiple stress resistance in sesame. In this study, NAC transcription factors were analyzed in sesame and some specific candidate *SiNAC* genes in response to abiotic stress for functional study were identified. This study provides valuable information to deepen our understanding of the abiotic stress responses by NAC transcription factors in sesame.

## Introduction

Sesame (*Sesamum indicum*), one of the most important oilseed crops worldwide, has long been considered as a representative health food in China, Korea, Japan, and other East Asian countries [1]. The yield and quality production of sesame is seriously compromised by multiple detrimental environmental factors, such as waterlogging, drought, salinity, pests, and diseases. Transcription factors (TFs) are types of regulatory proteins that control plant growth and development, as well as their responses to environmental changes, by regulating the expression of downstream genes [2–4]. For example, it has been demonstrated that some MYB TFs play significant roles in different biological processes, such as hormone signal transduction, organ development, metabolism, and responses to biotic and abiotic stresses [5–7]. Expression profiles analysis in different species have shown that many WRKY genes are involved in responses to various abiotic stresses, like drought, waterlogging, wounding, and salt stress [8–11]. The identification and functional characterization of TFs is a major area of transcription machinery research. The release of a high-quality genome sequence of sesame offers an excellent research opportunity to genome-wide analysis of sesame gene family [12].

The NAC (NAM, ATAF, and CUC) domain protein family is one of the largest plant-specific TF families. Its name was originally derived from the three proteins that contain a similar DNA-binding domain: no apical meristem (NAM), ATAF1-2, and cup-shaped cotyledon (CUC) [4, 13]. Typically, NAC proteins possess a conserved NAM domain at the N-terminus and a divergent transcription regulatory region at the C-terminus [14]. The N-terminal NAC domain usually comprises nearly 160 amino acid (aa) residues that are divided into five subdomains: A—E [14]. Subdomains A, C, and D are commonly highly conserved, whereas subdomains B and E are highly divergent and might confer functional diversity to NAC TFs [3, 14]. Subdomain A may play an important role in the formation of functional dimers with other NAC domain proteins, and subdomains C and D bind to DNA. In contrast, the C-terminal transcription regulatory regions are highly divergent and operate as functional domains by conferring regulation diversity of transcriptional activation activity [3, 14]. Moreover, some NAC TFs contain transmembrane motifs at the C-terminal end, which function in plasma membrane or endoplasmic anchoring [15].

NAC was firstly identified in *Arabidopsis thaliana* as a key gene for pattern formation in embryo and flowers in *Petunia* [13] and organ separation [4]. Later, it was found that NAC proteins regulated a variety of plant developmental processes, such as the formation of adventitious shoots [16], development of the shoot apical meristem [4, 13], flower development [17], leaf senescence [18], lateral root development [19], floral morphogenesis [20], cell cycle control [21, 22], hormone signaling [19, 21, 23], and grain nutrient remobilization [24]. Although the exact regulatory mechanism of NAC genes is not much known, their activity can be regulated through different processes: binding of specific TFs to NAC regulatory region in the promoter at transcriptional level; miRNA164-mediated cleavage of NAC genes at post-transcriptional level; NAC protein degradation mediated by ubiquitins, dimerization and interaction with other non-NAC proteins at post-translational level. In recent years, increasing evidences have shown that a large number of NAC genes play important roles in the regulation of plant tolerance to abiotic stresses [3]. In *Arabidopsis*, *AtNAC2*, *AtNAC3*, *ANAC019*, and *RD26* regulate the expression of stress-responsive genes involved in multi-abiotic stress tolerance [25–28]. Transgenic *Arabidopsis* overexpressing *ATAF1*, *MlNAC9*, or *CarNAC4* and transgenic rice overexpressing *SNAC1* or *OsNAC2* exhibits enhanced resistance to salt, drought and other stresses, respectively [29–33]. It was reported that some NAC membrane-bound TFs (NTLs) are mostly regulated in protecting plants against abiotic stresses [18, 21, 34]. For example, *Arabidopsis NTL4* is a membrane-bound NAC transcription factor which promotes reactive oxygen species production to during drought stress [11]. In Soybean, the heteroexpression of active membrane-bound *GmNTL1/GmNLT11* proteins was found to cause improved tolerance to abiotic stresses [35]. All these studies indicate that specific NAC TFs are very important in plants’ physiological signaling and multi-abiotic stress tolerance as well as plant development.

Recently, 117 NAC genes in *Arabidopsis* [36], 74 in grape (*Vitis vinifera*) [37], 163 in *Populus trichocarpa* [38], 152 in *Nicotiana tabacum* [39], 151 in *O*. *sativa* [36], 101 in *Brachypodium distachyon* [40], and 152 in *Zea mays* [41]were identified by genome-wide analyses, respectively. Sesame is one of the most important oil crop plants and affected seriously by multiple biotic and abiotic stresses [42]. Although a lot of NAC TFs have been functionally described in *Arabidopsis*, *O*. *sativa*, and other plants, only one sesame NAC gene (*SiNST1*/*SiNAC58*, *SIN_1005755*) was found significantly associated with content of lignin and seed coat thickness [43]. The functions of most of NAC members in sesame were unknown. In this study, we performed a comprehensive investigation of the NAC gene family in sesame through their identification, molecular characterization, phylogenetic analysis, and expression profiling in different tissues (root, stem, leaf, flower, seed, and capsule) and various abiotic stresses (drought, waterlogging, cold, osmotic, and salt). The results may lay the foundation for future functional characterizations of NAC TFs in sesame.

## Results

### Identification of NAC members in sesame

To identify the NAC family members in sesame, both Hidden Markov Model (HMM) and BLASTP searches were performed in the sesame genome with *Arabidopsis* and *O*. *sativa* NAC sequences as queries (S1 Table). A total of 87 putative NAC TFs with a conserved NAM domain were found in the complete sesame genome (S2 Table). Owing to the lack of a designated standard annotation for the 87 NAC genes in sesame, we named them *SiNAC01*–*SiNAC87* based on their position from top to bottom in the sesame linkage groups (LGs). The NAC genes identified in sesame encoded proteins ranging from 130 (*SiNAC56*) to 631 (*SiNAC85*) aa residues in length, with an average of 345 aa (S2 Table). Detailed information of sesame NAC genes, including accession numbers, precise positions, and similarities to their *Arabidopsis* orthologs are listed in S2 Table.

### Unequal distribution and gene duplication of *SiNAC* genes in the sesame genome

**Fig 1:**
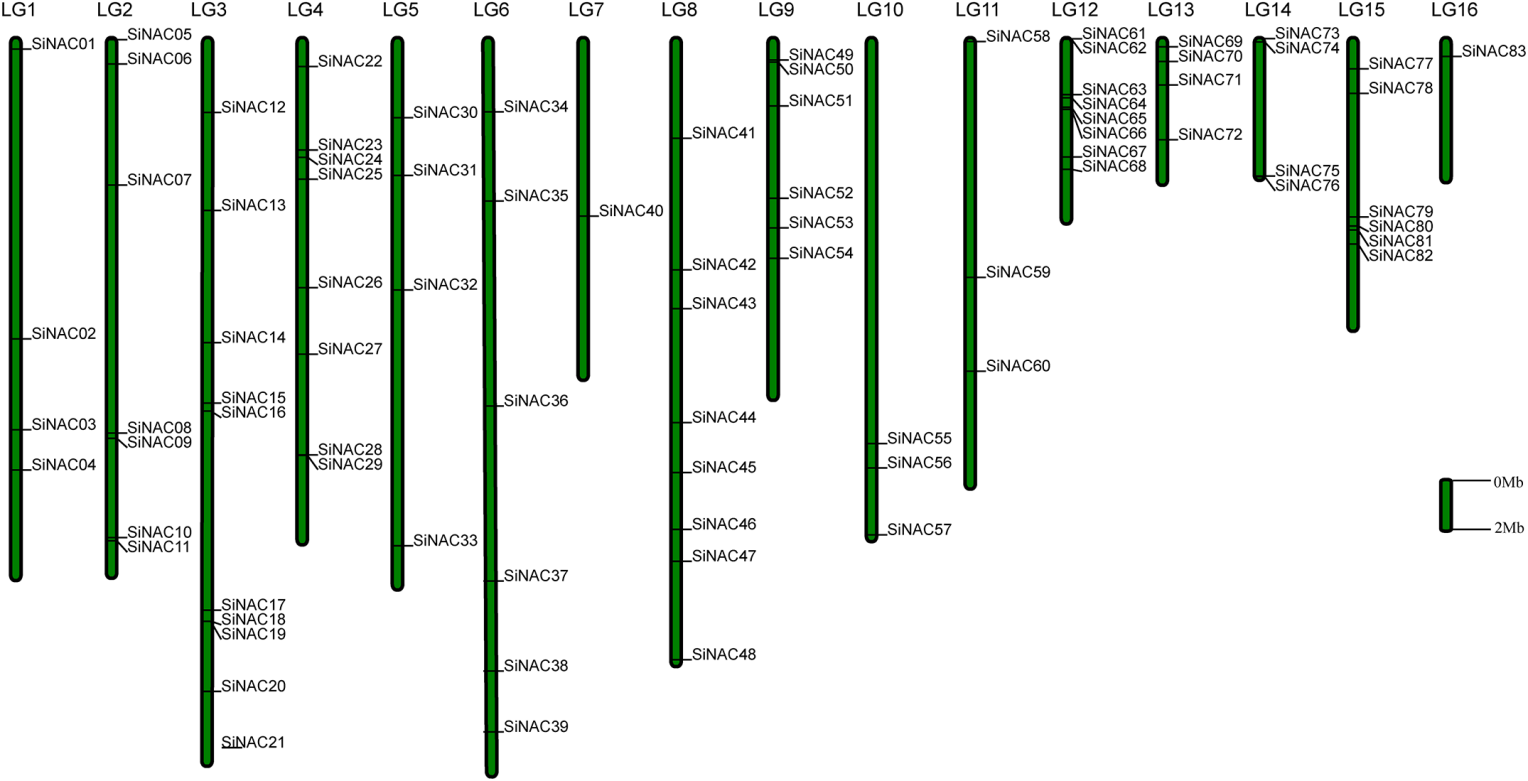
Distribution of *SiNAC* genes on 16 linkage groups (LGs). Vertical bars represent the LGs of sesame. The LG number is on the top of each LG. The scale is in 2 Mb.

A total of 83 members of the *SiNAC* gene family were distributed non-randomly on the 16 LGs in sesame, and four *SiNAC* genes (*SiNAC84–SiNAC87*) were mapped to unanchored scaffolds (Fig 1, S2 Table). LG 3 contained the largest number (10, ~11.5%) of sesame NAC genes, followed by LG 4, LG 8, and LG 12, each with eight members (~9.2%). In contrast, LG 7 and LG 16 contained only one gene each.

Four and 49 *SiNAC*s were found to be tandemly and segmentally duplicated, respectively which might have contributed significantly to the expansion of the *SiNAC* family (S2 Table). The 49 *SiNAC*s were located on duplicated segments on LGs 1–15 as illustrated in S1 Fig. The duplicated segments on LG 8 contains the highest number of *SiNAC*s (8), followed by six genes on the duplicated segments located on LG 12. In contrast, only one *SiNAC* each was found on the duplicated segments located on LGs 1, 7, and 13 (S1 Fig). The syntenic analysis of sesame, *Arabidopsis* and *V*. *vinifera* subgenomes showed 13 sesame NAC orthologous gene pairs between sesame and *Arabidopsis*, and 34 pairs between sesame and *V*. *vinifera* (S2 Fig, S2 and S3 Tables).

### Conserved motifs and gene structure of *SiNAC*s

**Fig 2:**
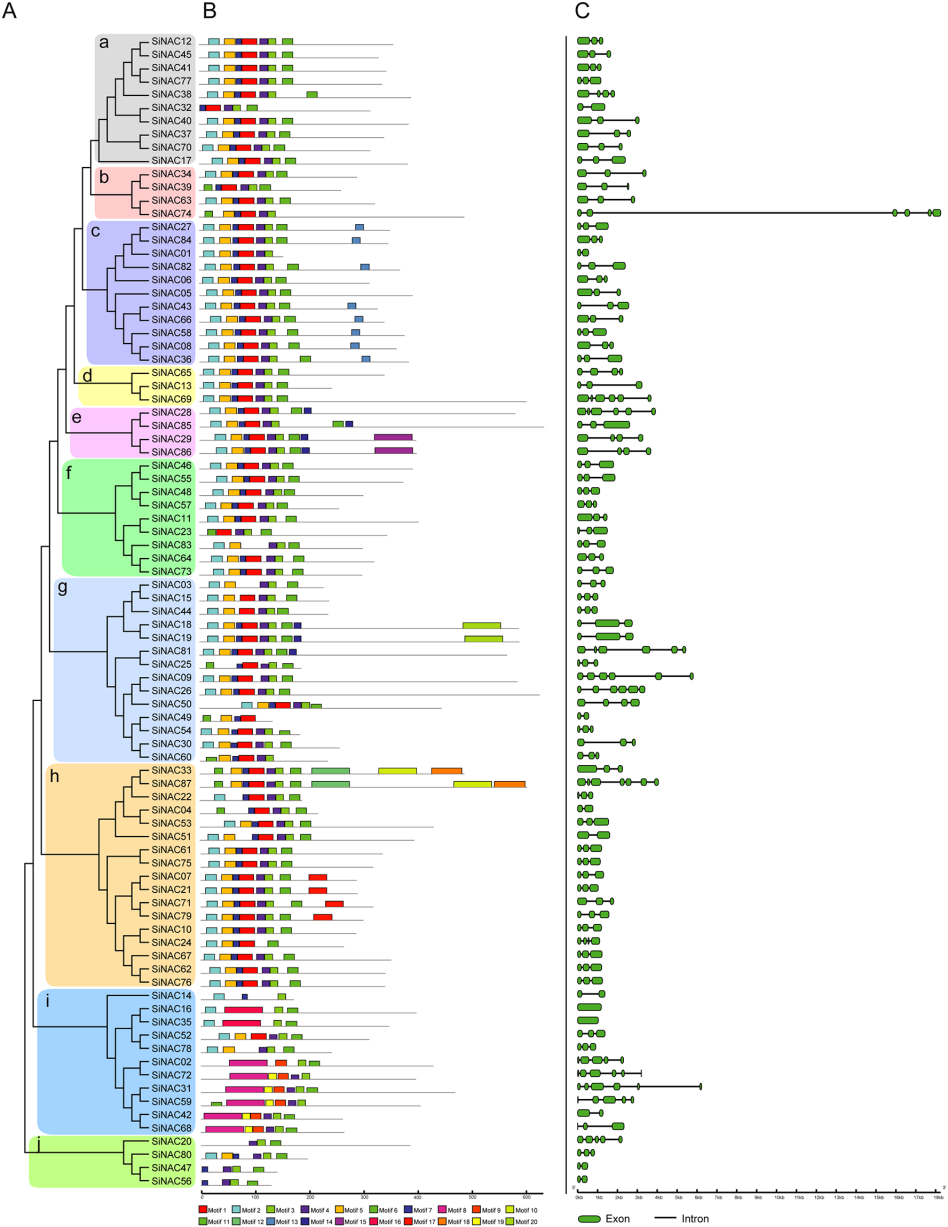
Phylogenetic relationships, motif compositions, and gene structure of *SiNAC* TFs. **(A)** The phylogenetic tree. The amino acid sequences of the SiNAC were aligned using ClustalX 2.1, and the phylogenetic tree was generated using MEGA 5.0 by the neighbor-joining method with 1000 bootstrap replicates. (**B)** Schematic representation of the conserved NAC proteins motifs from sesame elucidated by MEME. The colored boxes indicate the motifs. The black lines indicate the non-conserved sequences. The scale bar represents 100 aa. (**C)** Intron/exon structures of SiNAC genes. The black lines and green boxes indicate introns and exons, respectively. The scale bar represents 1.0 kb.

Multiple sequence alignment of sesame NAC proteins revealed that all SiNAC proteins included a highly conserved N-terminal NAC region (S3 Fig). Most of the SiNACs had a complete NAC domain consisting of five subdomains: A, B, C, D and E. However, *SiNAC32*, *SiNAC47*, and *SiNAC56* lacked A and B subdomains, and *SiNAC49* lacked D and E subdomains. To study the evolutionary relationship of these *SiNACs*, a neighbor-joining (NJ) tree was created from alignments of 87 NAC protein full-length sequences. The *SiNAC* TFs were divided into 10 groups, designated as subgroups a—j (Fig 2A). The subgroup h was the largest one, containing 17 members, whereas the subgroup d had only three members. In addition, most of the duplicated NAC members were clustered in the same subgroup. For instance, two duplicated NAC genes (*SiNAC08* and *SiNAC36*) were assigned to the subgroup c, and the other three (*SiNAC30*, *SiNAC54*, and *SiNAC60*) were classified into the subgroup g with high bootstrap values. The MEME program was used to predict putative motifs to get a better understanding of the diversity of NAC TFs from sesame. In total, 20 distinct motifs were identified (Fig 2B and S4 Fig). It is obvious that most of the closely related members in the phylogenetic tree exhibited common motif compositions, suggesting that the NAC members clustered in the same subgroup may have similar biological functions. The motif distribution analyses of the NAC proteins revealed that the N-termini of most *SiNAC* TFs contained five highly conserved subdomains (A—E), which conferred DNA-binding activity [14], except for most members of subfamily j which had no subdomains A and B (Fig 2B). In some specific subgroups, some conserved motifs were identified in C-terminal regions, such as motifs 10 and 18 in subgroup h and motif 15 in subgroup e (Fig 2B), suggesting that the specific functions of different subgroups could be due to specific motifs.

The exon/intron organization in the *SiNAC*s coding sequences were compared using online Gene Structure Display Server 2.0 (http://gsds.cbi.pku.edu.cn/). The result revealed that sesame NAC genes contain 0 to 6 introns, and the majority of *SiNAC* genes contained three exons. The detailed gene structure is presented in Fig 2C and S4 Table. Among these *SiNAC*s, those in most subfamilies harbored 2–5 introns, with the exception of *SiNAC16* and *SiNAC35* which had no intron. Overall, *SiNAC*s in the same phylogenetic group shared highly similar exon/intron structure including intron number, intron phase, and exon length.

### Phylogenetic analysis of *SiNAC*s

**Fig 3:**
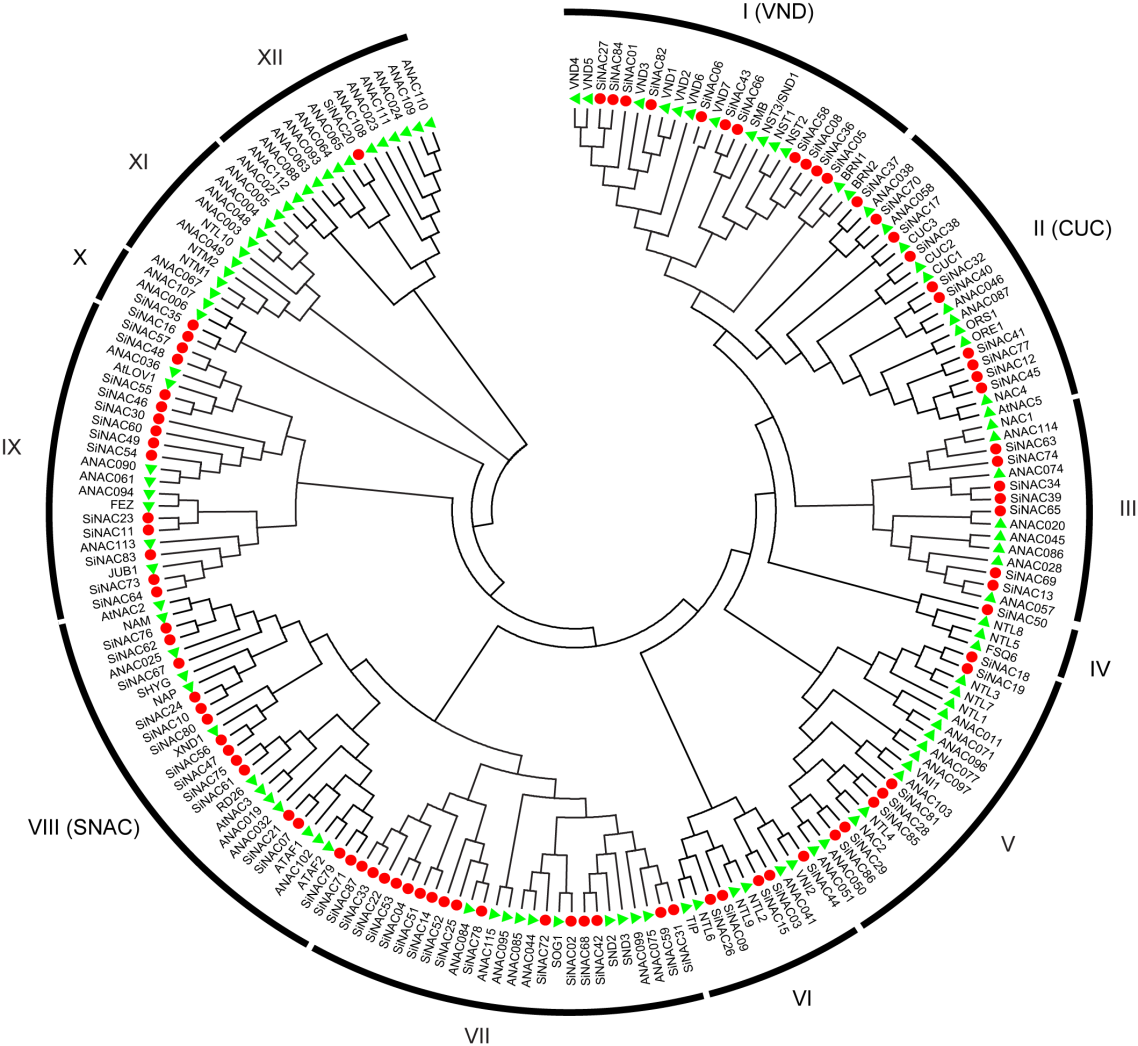
Phylogenetic tree of NAC proteins from sesame and *Arabidopsis*. The amino acid sequences of the NAC proteins were aligned using ClustalX 2.1, and the phylogenetic tree was generated using the neighbor-joining method with 1000 bootstrap replicates in the MEGA 5.0. The red dots and green triangles represent sesame and reported *Arabidopsis* NACs, respectively.

To explore the evolutionary relationships of NAC proteins from sesame and *Arabidopsis*, a NJ phylogenetic tree was created using full protein sequences of 203 NACs from sesame and *Arabidopsis*. The 87 *SiNAC* proteins formed 12 clades together with NACs from *Arabidopsis*, and were designated as subgroups I—XII (Fig 3). The largest clade was subgroup VII, which contained 16 *SiNAC* members; whereas the smallest was subgroup XII, with only one member. Subgroup XI contained only members from Arabidopsis, which implies that the homologs of these genes may have been lost in sesame following the divergence during the evolutionary process. Remarkably, the *Arabidopsis* NAC genes with the same functions exhibited a tendency to cluster into the same subgroup. For example, the VNDs (VND1-VND7) and the well-characterized NAC TFs involved in shoot organ boundary delimitation (CUC1, CUC2, and CUC3) were mainly located in subgroup I and II, respectively. Subgroup VIII encompassed many well-known stress-responsive *Arabidopsis* NAC genes, including *ANAC019*, *AtNAC2*, *AtNAC3*, *ATAF1*, *ATAF2*, and *RD26*. Therefore, these three subgroups were named according to their orthologous groups in *Arabidopsis*–VND (secondary wall synthesis NAC, subgroup I), CUC (development-related NAC, subgroup II), and SNAC (stress-related NAC, subgroup VIII) (Fig 3).

### Membrane-bound SiNAC subfamily

**Fig 4:**
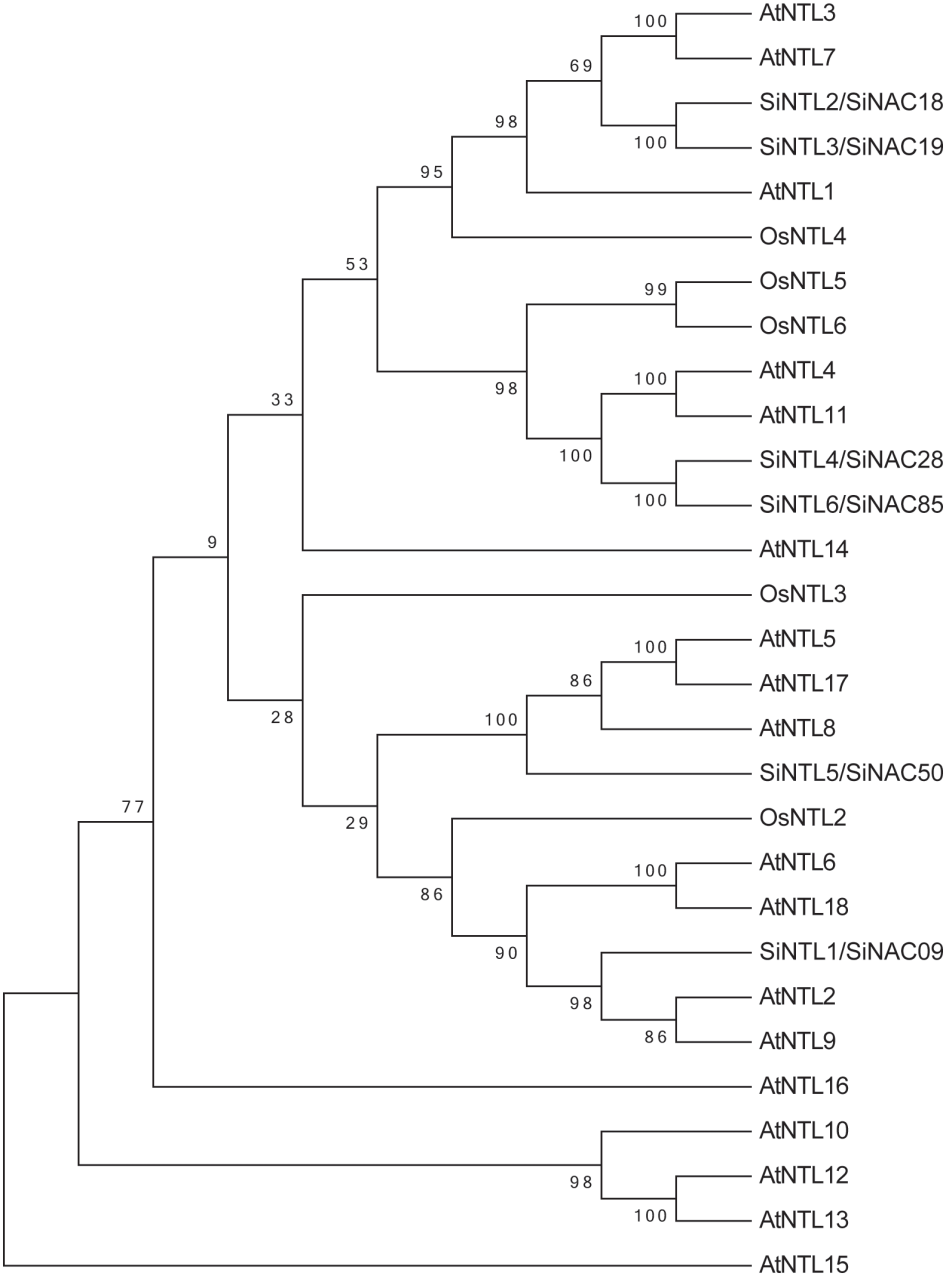
Phylogenetic relationships of membrane-bound NACs of sesame, *Arabidopsis*, and *O*. *sativa*. The full-length amino acid sequences of MTFs were aligned by Clustal X 2.1, and the phylogenetic tree was constructed using MEGA 5.0 by the neighbor-joining method with 1000 bootstrap replicates.

Recently, NAC membrane-bound TFs (MTFs) have been identified in many plants and implicated in response to abiotic stress [18, 21, 34]. In the present study, six SiNAC proteins containing α-helical transmembrane motifs at C-terminal ends were identified using the SMART web server, and named SiNTL1–SiNTL6 (Table 1). A NJ phylogenetic tree of NAC MTFs from sesame, *Arabidopsis*, and *O*. *sativa* was performed (Fig 4). The phylogenetic tree revealed that sesame MTFs had a close relationship with *Arabidopsis* MTFs suggesting similar functions of these genes in both species (Fig 4).

**Table 1:** Putative membrane-bound NAC transcription factors in sesame.

Name	Gene symbol	Locus ID	Size (aa)	Transmembrane regions
SiNTL1	*SiNAC*09	SIN_1021174	583	556–578
SiNTL2	*SiNAC*18	SIN_1015779	585	561–583
SiNTL3	*SiNAC*19	SIN_1015778	586	560–582
SiNTL4	*SiNAC*28	SIN_1003345	578	550–572
SiNTL5	*SiNAC*50	SIN_1010589	443	417–439
SiNTL6	*SiNAC*85	SIN_1001279	631	608–630

### Expression profiles of *SiNAC* genes in different tissues of sesame

**Fig 5:**
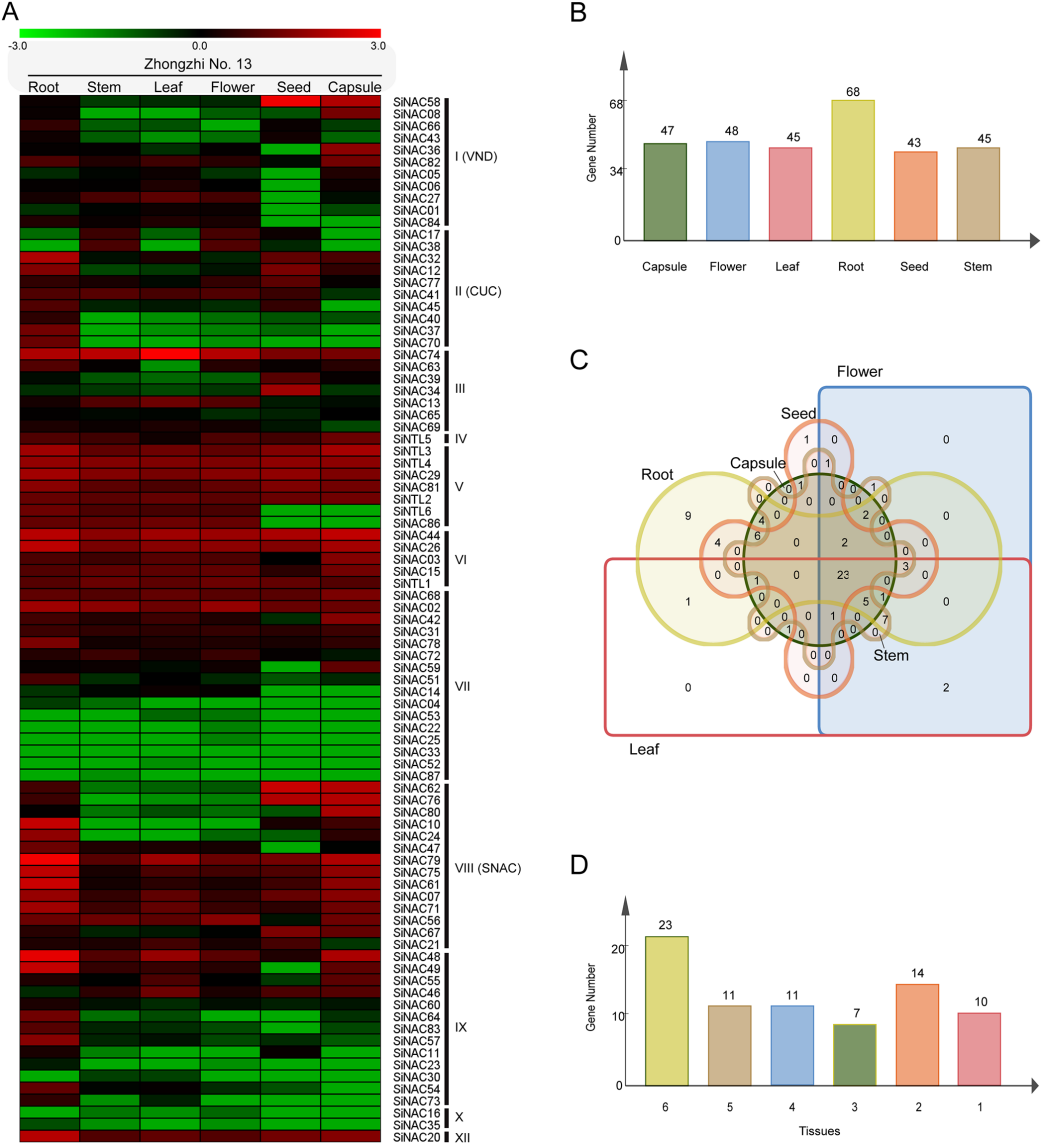
Expression patterns of *SiNAC* genes in different tissues. (**A)** Hierarchical clustering of expression profile of *SiNAC* genes in different tissues. Log_10_-based RPKM values were used to create the heat map with clustering. The relative signal intensity of RPKM values was represented The scale. (**B)** Number of genes specifically in each tissue. **(C)** An overview of *SiNAC* gene numbers in six tissues. **(D)** Number of genes: specific (1) or shared by 2, 3… tissues.

The expression profiles of *SiNAC* genes from six tissues, including root, stem, leaf, flower, seed, and capsule of cv. Zhongzhi No. 13 under normal growth conditions were investigated using transcriptomic data from sesameFG (http://www.ncgr.ac.cn/SesameFG)[44]. Transcript abundance was determined by use of reads per kilobase per million mapped reads (RPKM). Heatmap representing the transcriptomic data showed that 78.2% (68/87), 51.7% (45/87), 51.7% (45/87), 55.2% (48/87), 49.4% (43/87), and 54.0% (47/87) of *SiNAC* genes were expressed (RPKM value > 1) in root, stem, leaf, flower, seed, and capsule, respectively (Fig 5A and 5B). Moreover, 23 *SiNAC* genes were expressed (RPKM value > 1) in all organs, and 11, 11, 7, 14, and 10 *SiNAC* genes were expressed (RPKM value > 1) in five-, four-, three-, two-, and one-organ, respectively (Fig 5C and 5D). Nine *SiNAC* genes (*SiNAC37*, *SiNAC40*, *SiNAC51*, *SiNAC57*, *SiNAC60*, *SiNAC64*, *SiNAC70*, *SiNAC73*, and *SiNAC83*) were specifically expressed (RPKM value > 1) in root and only *SiNAC34* was specifically detected in seed (Fig 5A and 5C). *SiNAC58*, *SiNAC62*, *SiNAC67*, and *SiNAC76* were expressed (RPKM value > 10) in both seed and capsule (Fig 5A). In addition, many *SiNAC* genes from the same phylogenetic subgroup shared similar gene expression profiles in different tissues of sesame, especially some duplicated *SiNAC* members, such as *SiNAC43* and *SiNAC66* (subgroup I), *SiNAC34* and *SiNAC39* (subgroup III), *SiNAC03*, *SiNAC15* and *SiNAC44* (subgroup VI), *SiNAC26* and *SiNTL1* (subgroup VI), *SiNAC10* and *SiNAC24* (subgroup SNAC), *SiNAC62*, *SiNAC67* and *SiNAC76* (subgroup SNAC) (Fig 5A). However, some duplicated *SiNAC* genes (*SiNAC07* and *SiNAC21*, *SiNAC12* and *SiNAC45*, *SiNAC30* and *SiNAC54*, *SiNAC32* and *SiNAC40*,etc.) showed different expression profiles in one or more tissues of sesame, suggesting that the function of these duplicated genes may have changed through the course of evolution in sesame. Overall, the organ expression profiles of *SiNAC* genes may provide a foundation for further study of sesame growth and development.

### Expression profiles of *SiNAC* genes in response to drought

**Fig 6:**
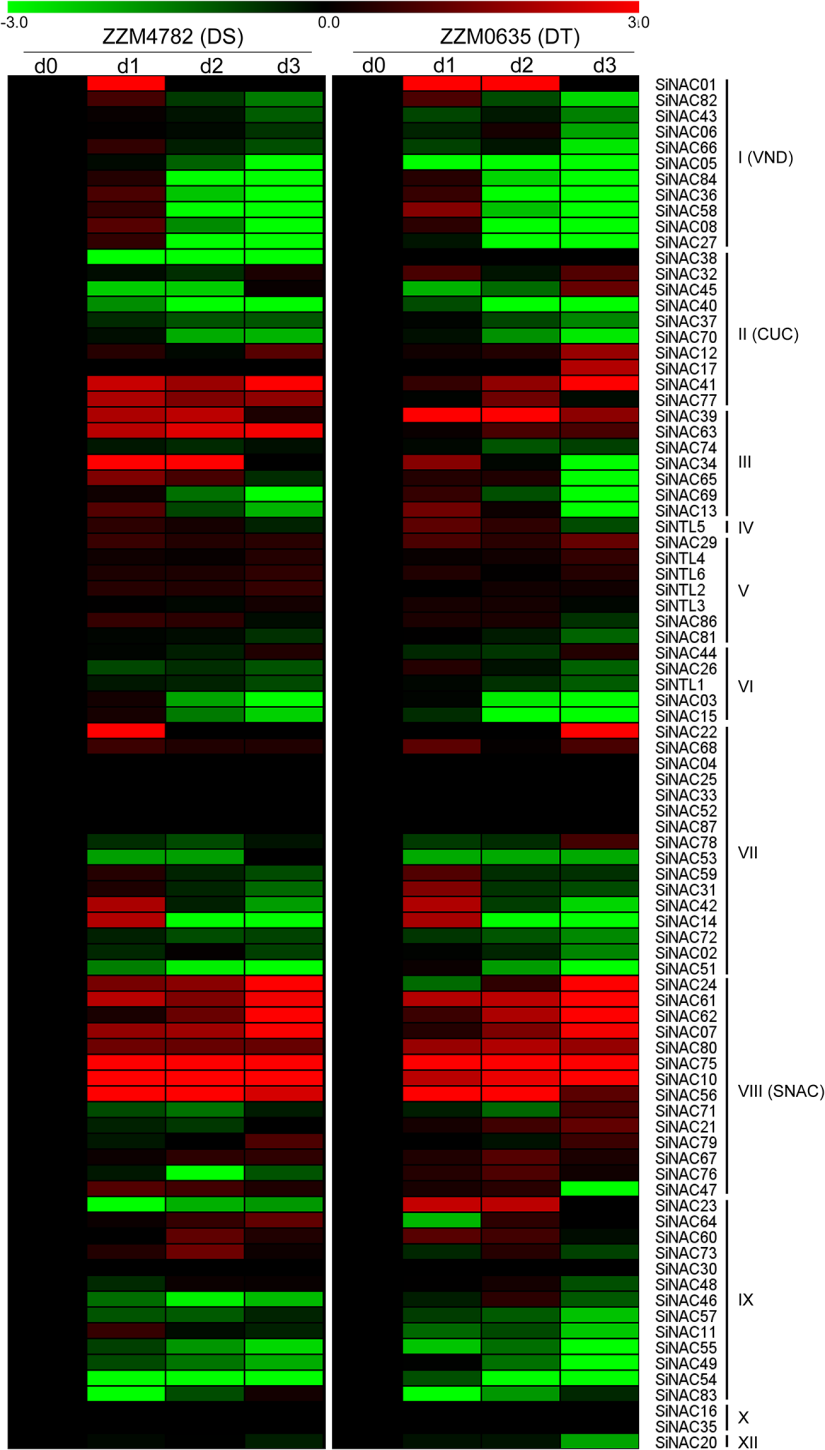
Expression profiles of *SiNAC* genes in roots of drought-tolerant (DT) and drought-sensitive (DS) cultivars after drought treatment. d0 (control), d1, d2, and d3 indicated the samples harvested with the soil water content at 35%, 15%, 9%, and 6%, respectively. Log_2_-transformed values of the relative expression levels of the *SiNAC* genes under drought stress were used to create the heat map. Changes in gene expression are shown in color as the scale.

To determine the expression patterns of *SiNAC* genes in response to drought, transcriptome data of drought-tolerant (DT) cv. ZZM0635 and drought-sensitive (DS) cv. ZZM4782 under drought stress were analyzed. When the soil water content at 35, 15, 9, and 6%–d0 (control), d1, d2, and d3 treatments, respectively—the root tissues of DT and DS plants were harvested for RNA-seq analysis [45]. A total of 79 *SiNAC*s common to DT and DS plants were expressed differentially after drought treatment (Fig 6). Of these, nine (*SiNAC07*, *SiNAC10*, *SiNAC39*, *SiNAC41*, *SiNAC56*, *SiNAC61*, *SiNAC62*, *SiNAC75*, and *SiNAC80*) were significantly up-regulated [log_2_ fold-change (FC) > 1] and nine (*SiNAC03*, *SiNAC05*, *SiNAC09*, *SiNAC15*, *SiNAC40*, *SiNAC49*, *SiNAC51*, *SiNAC54*, and *SiNAC55*) were down-regulated (log_2_FC < –1) in roots of both DT and DS plants at all the three time points during drought treatment (Fig 6). Many *SiNAC*s (especially some duplicated *SiNAC* genes) from the same phylogenetic subgroup showed similar expression profiles under drought stress between the DT and DS cultivars, suggesting that those SiNACs may have similar functions in the process of sesame response to drought (Fig 6). In particular, 8 *SiNACs* (*SiNAC07*, *SiNAC10*, *SiNAC24*, *SiNAC56*, *SiNAC61*, *SiNAC62*, *SiNAC75*, and *SiNAC80)* from the subgroup SNAC were identified to be significantly up-regulated in the roots of both DT and DS plants at least one time point during drought treatment. However, some genes showed different expression profiles between DS and DT cultivars. Expressions of *SiNAC21*, *SiNAC23*, *SiNAC32*, *SiNAC38*, *SiNAC46*, and *SiNAC76* were down-regulated in roots of DS plants, but increased or were unchanged in DT plants after drought treatment. These NAC genes responding to drought treatment may play important roles in regulation of sesame drought tolerance.

### Expression profiles of *SiNAC* genes under waterlogging stress

**Fig 7:**
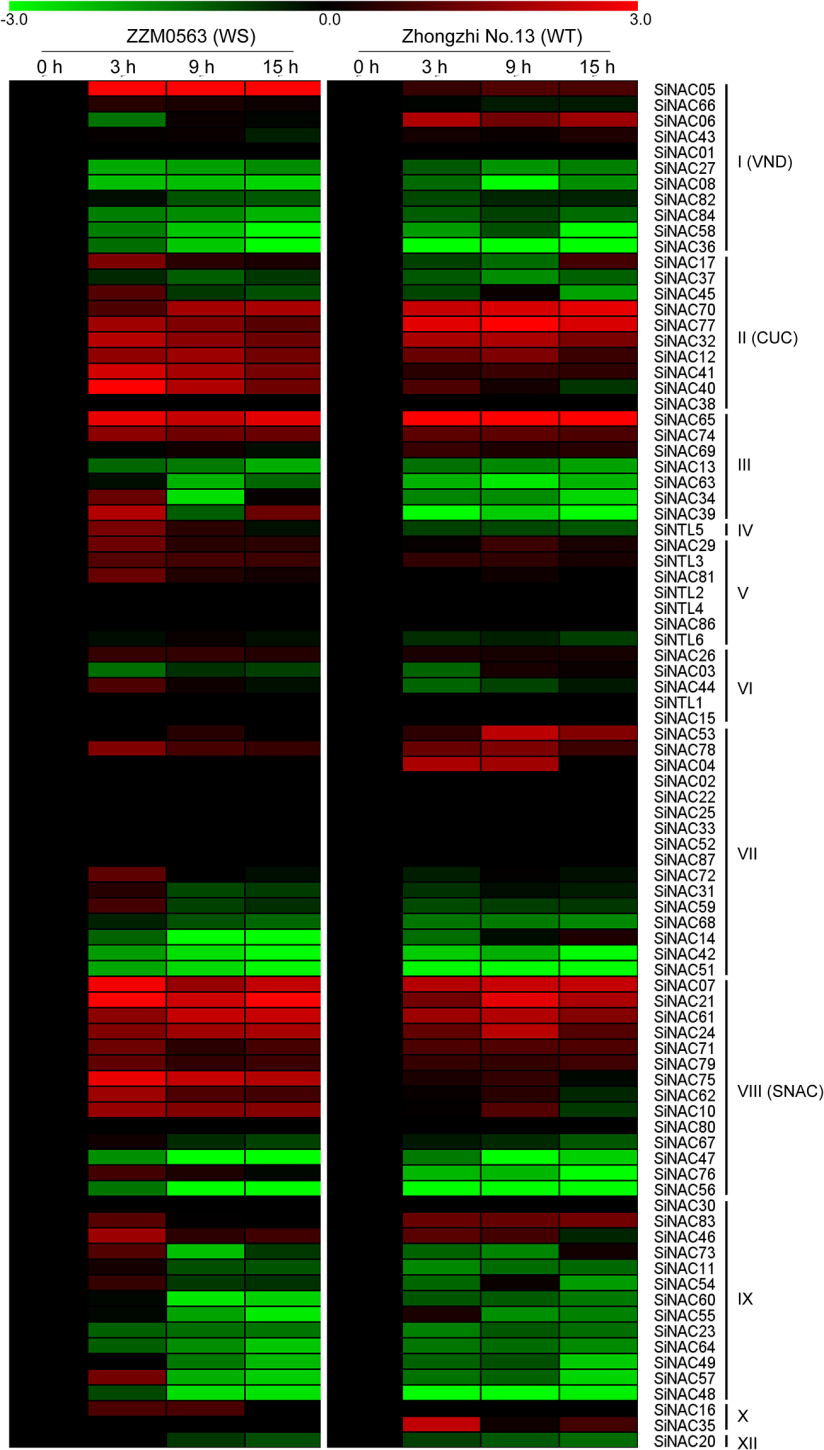
Expression profiles of *SiNAC* genes in roots of waterlogging-tolerant (WT) and waterlogging-sensitive (WS) cultivars after waterlogging treatment. Log_2_-transformed values of the relative expression levels of the *SiNAC* genes under waterlogging stress were used to create the heat map. Changes in gene expression are shown in color as the scale.

To determine the expression profiles of *SiNAC*s under waterlogging stress, the 87 NAC genes were analyzed using transcriptome data obtained from root samples of waterlogging-tolerant (WT) cv. Zhongzhi No. 13 and waterlogging-sensitive (WS) cv. ZZM0563 when waterlogged for 0, 3, 9, and 15 h [42]. Similar to the expression profiles in response to drought stress, most of the *SiNAC*s from the same phylogenetic subgroup also showed similar expression profiles in response to waterlogging stress between WT and WS genotypes (Fig 7). Among them, 11 *SiNAC*s were up-regulated (log_2_FC > 1) and 20 *SiNAC*s were down-regulated (log_2_FC < –1) in roots of both DT and DS plants for at least two time points during waterlogging treatment. Obviously, 3 *SiNAC* genes (*SiNAC12*, *SiNAC41*, and *SiNAC77*) of the subgroup II and 6 *SiNACs* (*SiNAC07*, *SiNAC10*, *SiNAC24*, *SiNAC61*, *SiNAC62*, and *SiNAC75*) of the subgroup SNAC were identified to be significantly up-regulated in the roots under drought and waterlogging treatments (Fig 7). However, the expression patterns of some genes between WS and WT plants differed. Notably, *SiNAC04*, *SiNAC06*, *SiNAC35*, *SiNAC53*, and *SiNAC83* were up-regulated in WT roots, but did not significantly change in WS plants after waterlogging treatment (Fig 7). In addition, *SiNAC10*, *SiNAC16*, *SiNAC17*, *SiNAC44*, *SiNAC50*, *SiNAC66*, *SiNAC72*, *SiNAC75*, and *SiNAC81* were up-regulated in WS roots, but detected down-regulated or unaffected in WT roots after waterlogging treatment (Fig 7). The functions of those waterlogging-responsive *SiNAC*s may enhance resistance of sesame in response to waterlogging stress.

### Expression profiles of *SiNAC* genes upon exposure to various abiotic stresses

**Fig 8:**
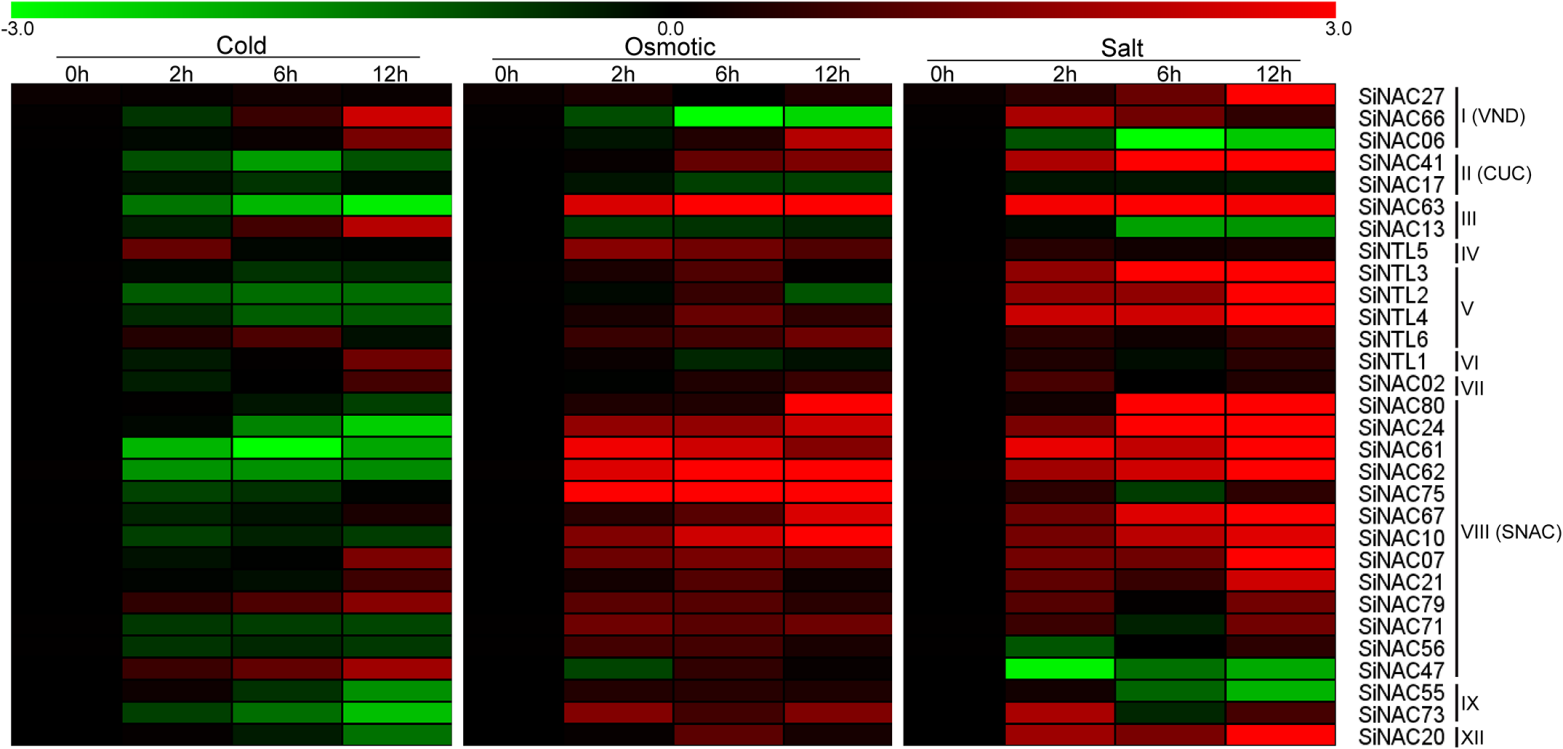
Expression profiles of *SiNAC* genes in leaves upon exposure to various abiotic stresses. 14-day-old seedlings were treated to osmotic (15% PEG 6000), salt (150 mM NaCl), and cold (4°C) stresses. Relative expression levels of the *SiNAC*s were analyzed by qRT-PCR, and the heat maps were created by log2-based fold-change values (mean of three biological replicates) (original data were shown in S5 Fig).

To extend our understanding of *SiNAC* genes in response to various environmental stresses, 30 *SiNAC*s (including all *SiNTL* genes, all members of subgroup SNAC, and one or two members of other subgroups) distributed in different subgroups were selected for further study of their responses to cold, osmotic, and salinity treatments at transcriptional level using quantitative real-time PCR (qRT-PCR). Heatmaps representation of expression FC in responses to the three stresses are shown in Fig 8 (original data were shown in S5 Fig). Under cold treatment, the majority of detected *SiNAC*s were down-regulated at two or three time points. Some *SiNAC*s were also induced by low temperature. Among them, *SiNAC13*, *SiNAC47*, *SiNAC66*, and *SiNAC79* were highly up-regulated with low-temperature condition (Fig 8). Most of the detected *SiNAC*s were up-regulated at all the three time points after osmotic treatment (Fig 8). Among them, *SiNAC75*, *SiNAC10*, *SiNAC62*, *SiNAC80*, and *SiNAC63* were highly up-regulated at 12 h after osmotic treatment (Fig 8). Notably, almost all of subgroup SNAC members were greatly sensitive to osmotic treatment, indicated that their functions in enhancing tolerance to abiotic stresses. After salinity treatment, the expression of most detected *SiNAC*s was up-regulated at two or three time points (Fig 8). Among them, expression of 15 genes was significantly up-regulated from 6 to 12 h suggesting that they may participate in sesame salt-stress tolerance. In contrast, *SiNAC06*, *SiNAC13*, *SiNAC47*, and *SiNAC55*, were down-regulated at least at two time points after salinity treatment (Fig 8).

The heat maps showed that all analyzed *SiNAC*s displayed variations of expression levels in responses to one or more stresses at the different time points after treatments. In addition, the majority of the detected *SiNAC*s were up-regulated under osmotic and salt conditions, but down-regulated under cold treatment (Fig 8). Notably, *SiNAC07*, *SiNAC21*, and *SiNAC79* were up-regulated after cold, osmotic, and salt treatments (Fig 8). Overall, those NAC genes responding to one or more treatments may play a significant role in the regulation of sesame tolerance to different abiotic stresses.

## Discussion

Sesame is an important oilseed crop and its growth and productivity are severely affected by abiotic stresses, particularly drought and waterlogging. The NAC gene family is one of the largest families of TFs and plays important roles in plant growth, development, and response to abiotic and biotic stresses. NAC TF genes have been identified and characterized in many species [14, 36, 37], but there is little information about this family in sesame. In this study, 87 NAC genes were identified in the sesame genome. The sesame NAC gene family (350 Mb, 87 NAC genes) is small compared with those of *Arabidopsis* (125 Mb, 117 NAC genes) and *O*. *sativa* (466 Mb, 151 NAC genes) [12, 36, 46, 47]. However, it is large compared with its closest related woody perennial *V*. *vinifera* (490 Mb, 74 NAC genes) [37, 48]. We speculate that more NAC genes were identified in the sesame genome may reveal that more need for these genes of sesame in transcriptional regulations. Gene duplication is one of the main driving forces in the genetic systems and evolution of genomic [49], and previous studies revealed that segmental duplication was the main method of NAC family expansion in several species [38]. In this study, 56.3% (49/87) of *SiNAC*s were found to be segmentally duplicated (S2 Table and S1 Fig), which might have greatly contributed to the expansion of the NAC family in sesame.

Molecular characterizations of SiNAC TFs revealed that motif compositions and gene structures differed among the subgroups; however, the NAC proteins that clustered in the same subgroup showed a similar motif composition and gene structure, which was in agreement with reports in *Arabidopsis*, *O*. *sativa*, and *V*. *vinifera* [14, 36, 37] (Fig 2). The diversity of conserved motifs and gene structures may also indicate that SiNAC TFs have diverse biological functions. Structurally similar NAC proteins within species were found to be functionally orthologous [36, 50]. When compared with the well-studied species *Arabidopsis*, an evolutionary relationship analysis of the NAC subfamily in sesame was performed. In this study, some SiNAC TFs which clustered in the same subgroup, especially shared similar gene expression profiles in different tissues of sesame or in response to various abiotic stresses, are likely to possess similar biological functions. For example, the *Arabidopsis* NAC proteins (NSTs and VNDs) of subgroup VND are involved in secondary wall synthesis [51]. Four SiNAC genes (*SiNAC08*, *SiNAC36*, *SiNAC58*, and *SiNAC82*) from the same subgroup VND were found highly expressed in the capsules, indicating that these genes may regulate secondary wall synthesis in sesame capsule. Furthermore, *SiNST1/SiNAC58* was found significantly associated with lignin content and seed coat thickness in sesame [43], which is consistent with the function of its *Arabidopsis* homologous gene *NST1* [50]. Subgroup CUC encompassed the NAC proteins involved in shoot organ boundary delimitation [16, 52] and subgroup SNAC includes NAC genes such as *AtNAC2*, *AtNAC3*, *ATAF1*, *RD26*, and *ANAC019*, which have been shown to be induced by abiotic stresses including drought and salinity [23, 25–28]. Consequently, phylogeny-based functional prediction helpful for SiNACs’ functional characterization and should be a focus in research on functions of sesame NAC genes in the future.

In this study, tissue specific and stress responsive *SiNAC*s were identified with available transcriptome sequencing data and qRT-PCR. The different expression patterns of *SiNAC*s provide vital information for determining the functions of SiNAC TFs. The transcriptome sequencing data from different tissues of cv. Zhongzhi No. 13 showed that 23 *SiNAC*s were expressed in all organs, and several were expressed specifically in different tissues, suggesting that these sesame NAC genes may have specific functional roles in sesame organ growth and development. Notably, *SiNAC58* was highly expressed in seeds and capsules, consistent with its function of increasing content of lignin and seed coat thickness [43]. Evidence suggests that NAC genes play significant roles in the regulation of responses to various abiotic stresses in plants [23, 25–27, 32]. In this study, we identified many sesame NAC genes induced by one or more abiotic stresses. Almost all members of the subgroup SNAC were responsive to various abiotic stresses. Especially, three *SiNAC*s (*SiNAC07*, *SiNAC21*, and *SiNAC79*) were up-regulated under drought, waterlogging, cold, osmotic, and salt treatments, implying their roles in multiple abiotic stresses resistance. *SiNAC61* and *SiNAC62* were highly expressed after drought and salt treatments and may function to improve the endurance of sesame to drought and salt stresses. In addition, we found some members of other subgroups to be highly active in abiotic stress response. For example, expressions of *SiNAC04* (subgroup VII), *SiNAC06* (subgroup I), *SiNAC53* (subgroup VII), and *SiNAC83* (subgroup IX) increased in WT plants but did not obviously change in WS plants at 9 h after waterlogging, indicating that these four *SiNAC* genes might play significant roles in sesame waterlogging tolerance.

Previous studies show that the dormant form of NTLs were activated by proteolytic cleavage once stimulated, and the activated TFs regulate the target genes expression after the degradation of cytoplasmic anchors [15, 34, 53]. It is known that some of *Arabidopsis NTLs*, like *NTM2*, *NTL4*, *NTL6*, *NTL8* and *NTL9*, involved in response to abiotic stresses [18, 54–57]. Phylogenetic analysis of sesame NTLs with *Arabidopsis* and rice NTLs (Fig 4) suggesting similar functions of these genes and provide a reference for investigation of putative functions of sesame NTLs in the future. In this study, the six detected *SiNTL* genes were induced rapidly by at least one type of stress treatment—waterlogging, drought, cold, osmotic, and salt stresses—consistent with previous reports [3, 18, 34]. Notably, *SiNTL2/SiNAC18*, *SiNTL3/SiNAC19*, and *SiNTL4/SiNAC28* (homology of *Arabidopsis AtNTL4*) were significantly up-regulated after salt-stress treatment, but down-regulated after cold treatment, implying that these *SiNTL*s are functionally associated with the salt and cold stress responses. Overall, the expression profiles of *SiNAC*s under various stresses suggest that different *SiNAC*s may be involved in various signaling pathways and stress responses, and that one *SiNAC* gene also involved in multiple abiotic stress responses and stress resistance regulation in sesame.

## Conclusions

In this study, 87 sesame NAC genes were identified and the distribution, basic classification, gene structure, and evolutionary characteristics of them were investigated. The expression profiles of *SiNAC*s in different tissues can assist in understanding the molecular basis sesame development and growth. Furthermore, analyses of the expression profiles of *SiNAC* following various abiotic stress treatments indicated that they are highly active in responses to drought, waterlogging, cold, osmotic, and salinity treatments, reveal that sesame NACs may represent convergence points of different signaling pathways. This work may provide a strong basis for future functional recearch of NAC TFs in responses to abiotic stresses in sesame.

## Methods

### Identification of the NAC gene family in sesame

First, to identify the putative NAC proteins in the sesame genome database (Sinbase) (http://ocri-genomics.org/Sinbase/), the HMM profile of the NAM domain (PF02365) gained from Pfam 26.0 (http://pfam.xfam.org/) was utilized by using HMM search (HMMER3.0) with an expected value (e-value) cut off of 1E-10 [58]. Next, the protein sequences of 116 published *Arabidopsis* NAC (ANAC) and 149 *O*. *sativa* NAC (ONAC) [14, 36] TFs were used in the BLASTP program against Sinbase with an e-value ≤ 1E-10 and amino acid sequence > 100 residues (S1 Table). Finally, all non-redundant putative NAC protein sequences identified from HMM and BLAST searches were manually used to confirm the existence of the NAC domain with the help of SMART (http://smart.emblheidelberg.de/) and InterProScan (http://www.ebi.ac.uk/Tools/InterProScan/) web server [59, 60].

### Physical positions and gene duplication analyses of NAC genes

The physical positions of *SiNAC*s were mapped to the 16 sesame LGs using a GFF file downloaded from the Sinbase database [12]. An in-house Perl script was then used to draw graphic NAC genes into the LGs of the sesame genome [61]. For nomenclature, the prefix ‘Si’ for *S*. *indicum* was added followed by NAC and numbered refer to its physical position from top to bottom on sesame LGs 1–16.

Genome and NAC sequences of *Arabidopsis* and *V*. *vinifera* were extracted from the TIGR database (http://www.tigr.org/tdb/e2k1/ath1/) and Genoscope (http://www.genoscope.cns.fr). MCScanX was employed to analyze segmental duplication in the sesame genome and collinear analysis among the sesame, *Arabidopsis*, and *V*. *vinifera* genomes with default parameters [62]. Members of the NAC gene family with segmental duplication and collinear analysis were retrieved from the above data sets for further analysis. Circos was used to construct the diagram [63].

### Phylogenetics analysis and molecular characterization

Clustal X 2.1 and MEGA 5.0 were used to construct NJ phylogenetic trees based on aa sequence of NAC members from sesame and *Arabidopsis* with 1000 bootstrap replicates [64, 65]. A MEME v4.10.1 utility (Multiple Em for Motif Elicitation, http://meme-suite.org/index.html) was used to display the motifs of NAC proteins from sesame [66]. The exon/intron organizations of sesame NAC genes were performed from Gene Structure Display Server (http://gsds.cbi.pku.edu.cn/)[67]. The membrane-bound *SiNAC* protein predictions were determined by SMART web server (http://smart.embl-heidelberg.de/)[59].

### Plant growth, stress treatment, and RNA extraction

The sesame seeds were selected from sesame germplasm provided by the Oil Crops Research Institute, Chinese Academy of Agricultural Sciences, Wuhan, China. Uniform seeds of sesame cv. Zhongzhi No. 13 were sterilized with 3% sodium hypochlorite and washed four times using sterile water. The seeds were germinated on tow filter papers with sterile water in an illuminated incubator using a 16/8 h light/dark cycle at 28°C. Three days later, seedlings were placed in half-strength Hoagland solution. For cold stress, 14-day-old seedlings were transferred to a illumination incubator at 4°C. For osmotic- and salt-stress treatments, 14-day-old seedlings were treated with 15% PEG 6000 and 150 mM NaCl, respectively. The leaves of treated seedlings were harvested at 0 (control or CK), 2, 6, and 12 h for assays. All samples were immediately placed in liquid nitrogen and stored at –80°C until use. Total RNA of seedlings was extracted using an EASYspin Plus kit (Aidlab, Beijing, China). The RNA of samples was reverse transcribed using a HiScript II 1st Strand cDNA Synthesis kit (Vazyme Biotech, Nanjing, China) with oligo (dT23) primer.

### Expression analyses of *SiNAC* genes

The expression pattern of *SiNAC* genes was analyzed using three groups of transcriptome sequencing data obtained earlier by our group. For the expression analysis of *SiNAC* genes in different tissues, the transcriptome data were obtained from root, stem, leaf, flower, seed, and capsule of cv. Zhongzhi No. 13 under normal growth conditions. For the transcriptome data of drought stress treatment, samples of the roots of DT cv. ZZM0635 and DS cv. ZZM4782 were harvested when the soil water content was at 35% (d0 controls), 15% (d1 treatment), 9% (d2 treatment), and 6% (d3 treatment) at the early anthesis stage [45]. To determine the expression of sesame NAC genes in response to waterlogging, transcriptome data were obtained from roots of WT cv. Zhongzhi No. 13 and WS cv. ZZM0563 at 0, 3, 9, and 15 h after applying waterlogging [42]. Transcript abundance was calculated by RPKM. The hierarchical cluster analyses and heat maps were generated by MultiExperiment Viewer using log-transformed RPKM values [68].

To study the expression profiles of sesame NAC genes in response to cold (4°C), osmotic (15% PEG 6000), and salinity (150 mM NaCl) stresses, qRT-PCR programs were performed on a LightCycler480 Real-Time PCR System using ChamQ^™^ SYBR^®^ qPCR Master Mix (Vazyme Biotech, Nanjing, China) according to the manufacturer’s instructions. Expression of 30 sesame NAC genes distributed in different subgroups were detected by qRT-PCR with three biological replications, and the sesame *Histone H3*.*3* gene (*SIN_1004293*) was used as an internal control [69]. The gene-specific primers are listed in S5 Table. Relative gene expression data were analyzed with the 2^–ΔΔCT^ method [69].

## Supporting information

S1 Fig Forty-nine segmental duplicated *SiNAC* genes on 16 linkage groups. Grey lines represent collinear blocks in whole sesame genome, and red lines represent duplicated SiNAC gene pairs.
(TIF)

S2 Fig Synteny between *NAC* genes in sesame, *Arabidopsis*, and *V*. *vinifera* genomes. The green bars indicated the LGs of sesame and the chromosomes of *Arabidopsis*, and *V*. *vinifera*. The numbers 01–16 indicate sesame genome LGs, Chr1–Chr5 represent the five Arabidopsis chromosomes, and chr1–chr19 represent the nineteen grape chromosomes. Black lines on the green bars represent the NAC gene locations on the LGs or chromosomes. Colored lines represent orthologous genes in sesame, *Arabidopsis* and grape.
(JPG)

S3 Fig Multiple sequence alignment of sesame NAC proteins. Multiple sequence alignment of NAC domain from 87 SiNACs. The NAC subdomains A-E are represented by black lines above the sequences.
(TIF)

S4 Fig Schematic diagram of NAC protein motifs in sesame. Motif 2, 8 and 11 represents the NAC subdomain A, motif 5, 8 and 16 represents the NAC subdomain B, motif 1, 7 and 16 represents the NAC subdomain C, motif 3 and 4 represents the NAC subdomain D, and motif 6 represents the NAC subdomain E.
(TIF)

S5 Fig Expression profile of *SiNAC* genes in response to various abiotic stresses. 14-day-old seedlings were treated to osmotic stress (15% PEG 6000), salt (150 mM NaCl), and cold (4°C) stresses. Relative expression levels of *SiNAC* genes were analyzed by qRT-PCR. The expression levels are normalized with respect to reference gene *Histone H3*.*3* (*SIN_1004293*) in different samples. Error bars indicate standard deviations (SD) based on three replicates.
(TIF)

S1 Table The accession numbers of NACs in *Arabidopsis*, *O*. *sativa*, and *V*. *vinifera*. (XLSX)

S2 Table The NAC transcription factor family in sesame. (XLSX)

S3 Table Orthologous NAC gene pairs in sesame and *V*. *vinifera*. (XLSX)

S4 Table Structure of NAC TFs in sesame. (XLSX)

S5 Table Primers used in qRT-PCR analysis. (XLSX)

## Abbreviations

ATAF: *Arabidopsis* transcription activation factor
CUC: Cup-shaped cotyledon
DS: drought-sensitive
DT: drought-tolerant
HMM: Hidden Markov Model
LGs: Linkage groups
NAC: NAM, ATAF1/2 and CUC2 transcription factor
NAM: no apical meristem
NJ: Neighbor-joining
NTL: NAC membrane-bound transcription factor
qRT-PCR: quantitative real-time RT-PCR
RPKM: Reads per kilobase per million mapped reads
TFs: Transcription factors
WS: Waterlogging-sensitive
WT: Waterlogging-tolerant
