# Supplementary figures and images for: Genome-wide identification and comprehensive analysis of the NAC transcription factor family in Sesamum indicum
Source: PLoS One. 2018 Jun 21;13(6):e0199262. doi: 10.1371/journal.pone.0199262 (PMC6013105; doi:10.1371/journal.pone.0199262)

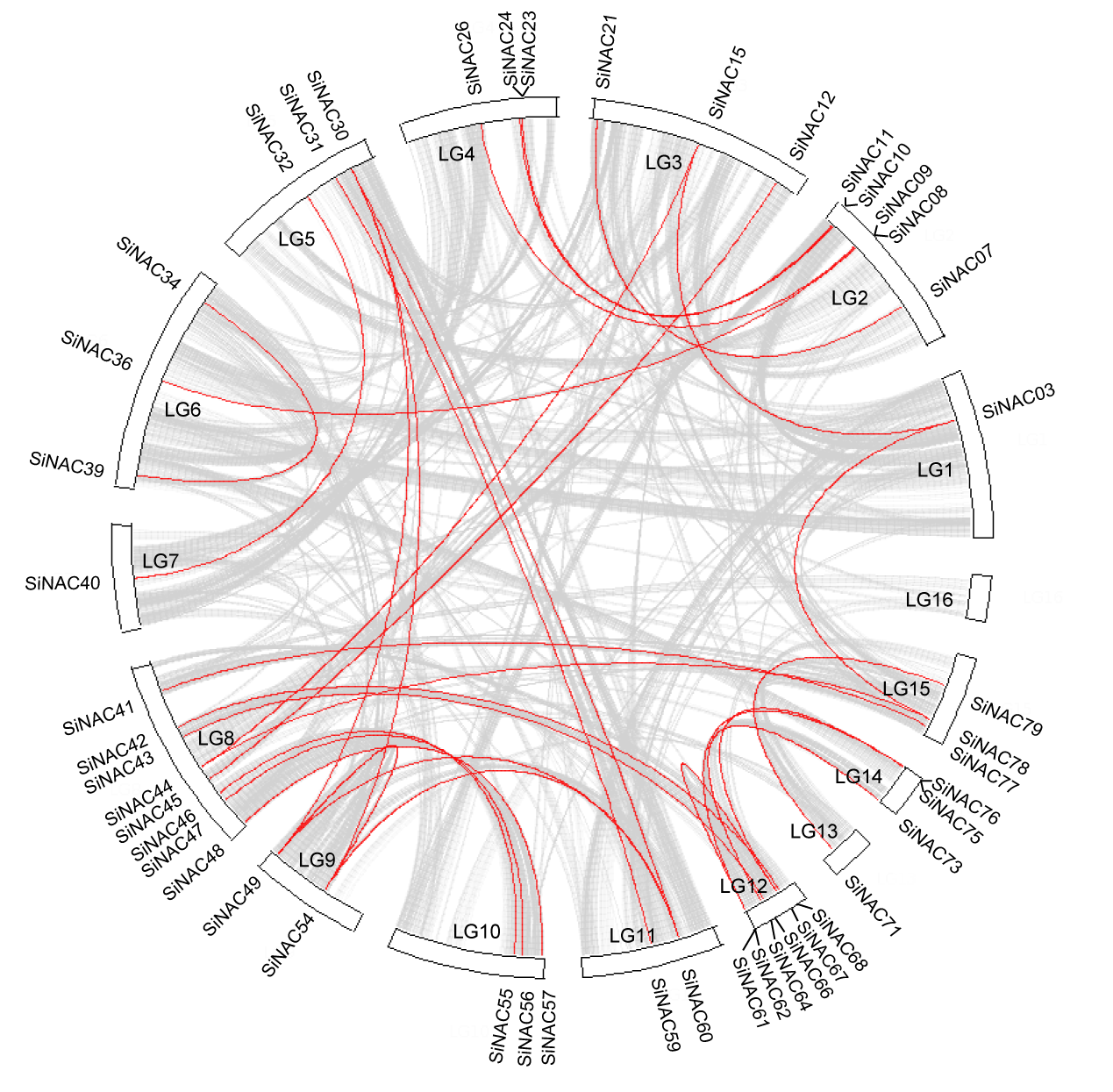

Supplement: S1 Fig — Grey lines represent collinear blocks in whole sesame genome, and red lines represent duplicated SiNAC gene pairs. (TIF) [file pone.0199262.s001.tif]

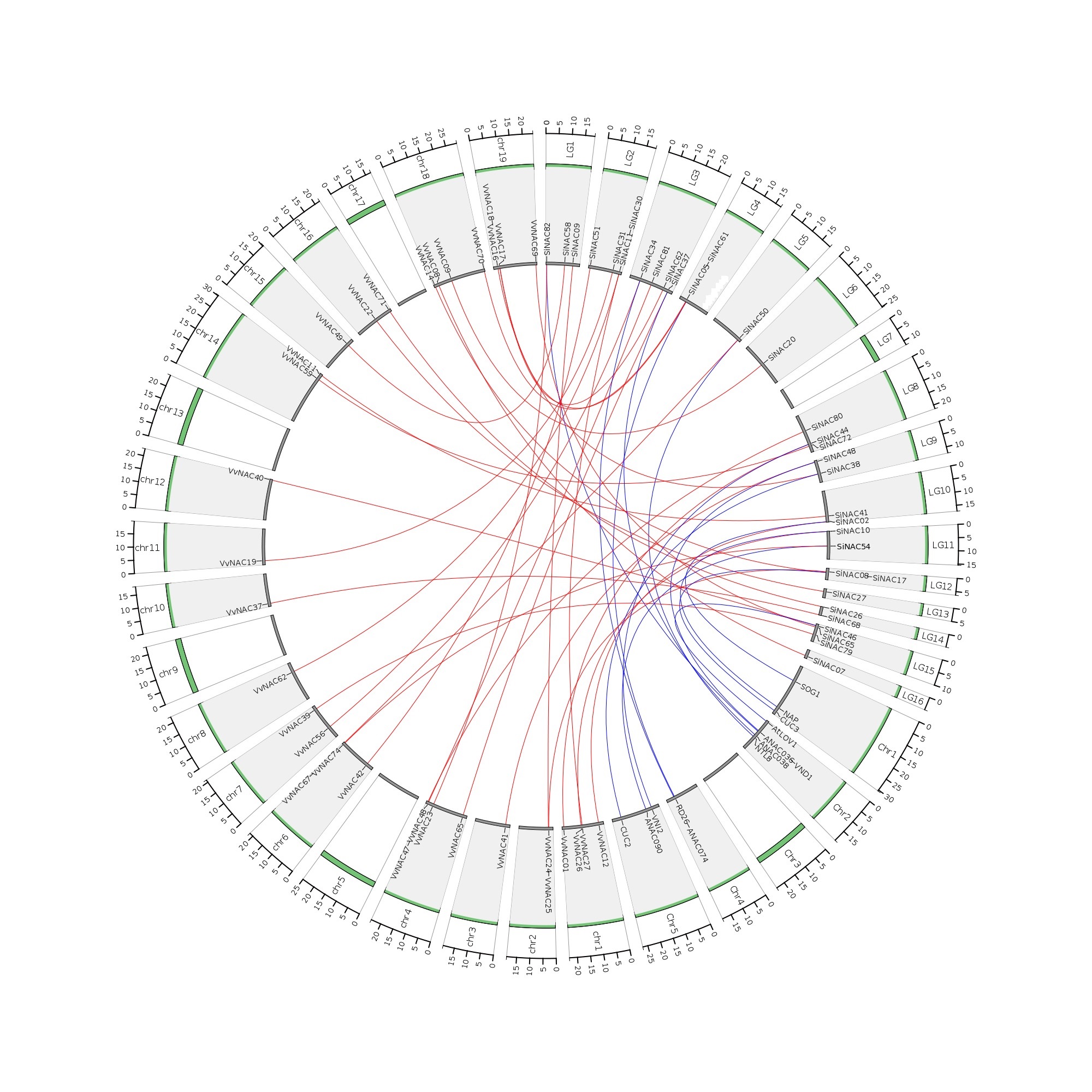

Supplement: S2 Fig — The green bars indicated the LGs of sesame and the chromosomes of Arabidopsis, and V. vinifera. The numbers 01–16 indicate sesame genome LGs, Chr1–Chr5 represent the five Arabidopsis chromosomes, and chr1–chr19 represent the nineteen grape chromosomes. Black lines on the green bars represent the NAC gene locations on the LGs or chromosomes. Colored lines represent orthologous genes in sesame, Arabidopsis and grape. (JPG) [file pone.0199262.s002.jpg]

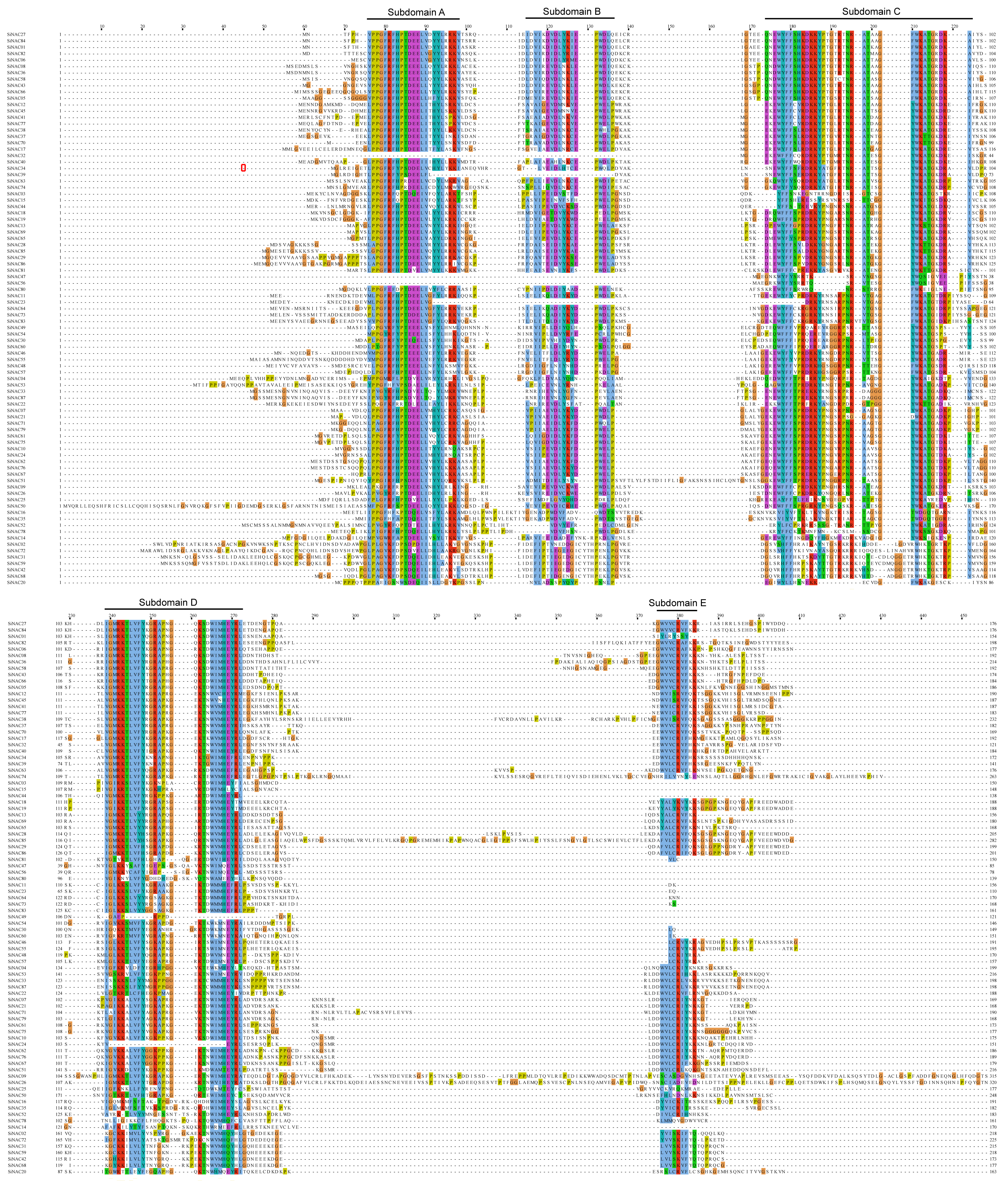

Supplement: S3 Fig — Multiple sequence alignment of NAC domain from 87 SiNACs. The NAC subdomains A-E are represented by black lines above the sequences. (TIF) [file pone.0199262.s003.tif]

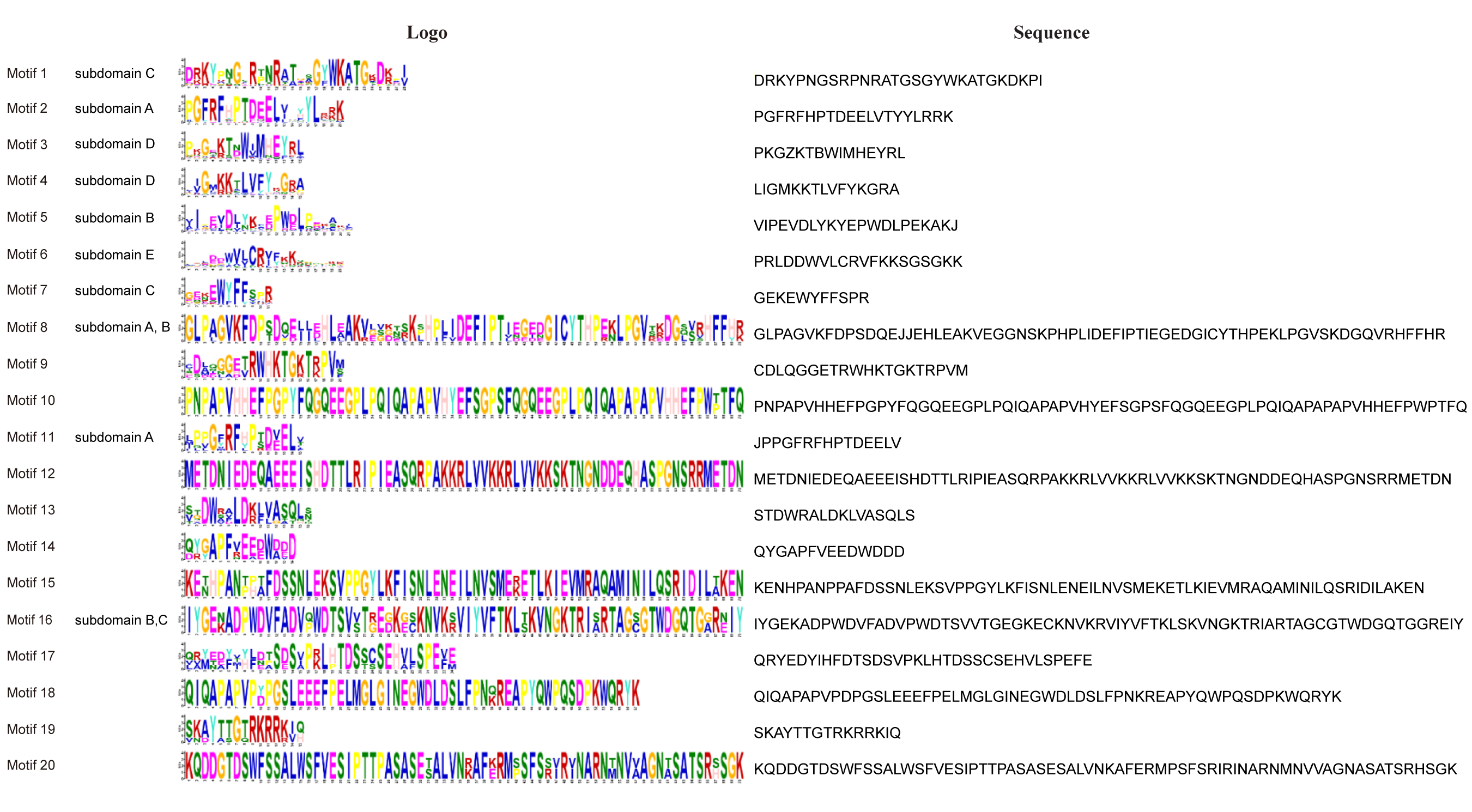

Supplement: S4 Fig — Motif 2, 8 and 11 represents the NAC subdomain A, motif 5, 8 and 16 represents the NAC subdomain B, motif 1, 7 and 16 represents the NAC subdomain C, motif 3 and 4 represents the NAC subdomain D, and motif 6 represents the NAC subdomain E. (TIF) [file pone.0199262.s004.tif]

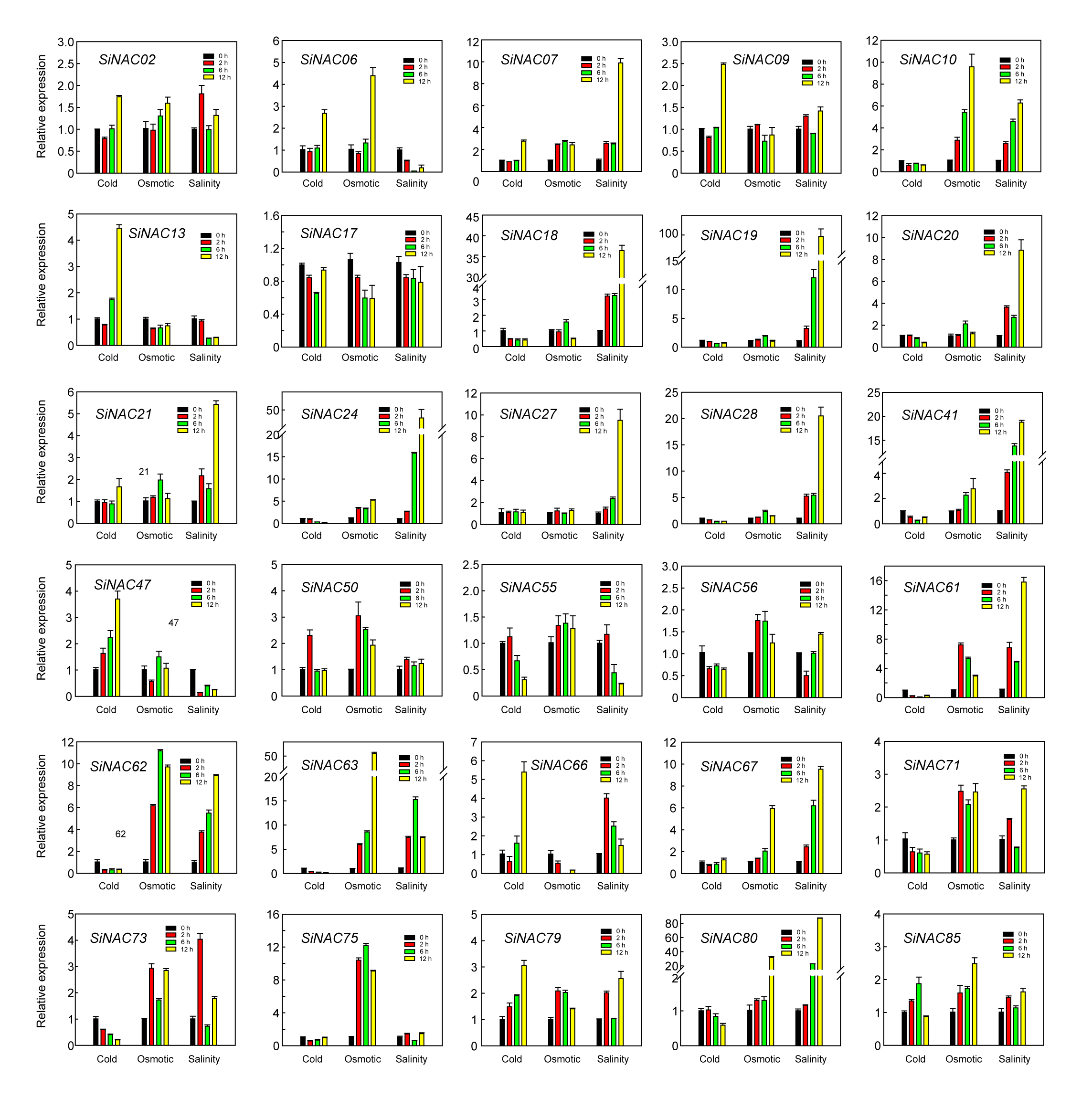

Supplement: S5 Fig — 14-day-old seedlings were treated to osmotic stress (15% PEG 6000), salt (150 mM NaCl), and cold (4°C) stresses. Relative expression levels of SiNAC genes were analyzed by qRT-PCR. The expression levels are normalized with respect to reference gene Histone H3.3 (SIN_1004293) in different samples. Error bars indicate standard deviations (SD) based on three replicates. (TIF) [file pone.0199262.s005.tif]
